# Heat Input Effect on Microstructure and Mechanical Properties of Electron Beam Additive Manufactured (EBAM) Cu-7.5wt.%Al Bronze

**DOI:** 10.3390/ma14226948

**Published:** 2021-11-17

**Authors:** Andrey Filippov, Nikolay Shamarin, Evgeny Moskvichev, Nikolai Savchenko, Evgeny Kolubaev, Ekaterina Khoroshko, Sergei Tarasov

**Affiliations:** 1 Institute of Strength Physics and Materials Science of the Siberian Branch of the Russian Academy of Sciences, Pr. Akademicheskiy 2/4, 634055 Tomsk, Russia; shamarin.nik@gmail.com (N.S.); em_tsu@mail.ru (E.M.); savnick@ispms.tsc.ru (N.S.); eak@ispms.ru (E.K.); eskhoroshko@gmail.com (E.K.); tsy@ispms.ru (S.T.); 2 National Research Tomsk State University, Lenin Av. 36, 634050 Tomsk, Russia

**Keywords:** additive manufacturing, heat input, bronze, transition zone, zigzagged columnar grains, tensile strength

## Abstract

Electron beam additive wire-feed deposition of Cu-7.5wt.%Al bronze on a stainless-steel substrate has been carried out at heat input levels 0.21, 0.255, and 0.3 kJ/mm. The microstructures formed at 0.21 kJ/mm were characterized by the presence of both zigzagged columnar and small equiaxed grains with 10% of Σ3 annealing twin grain boundaries. No equiaxed grains were found in samples obtained at 0.255 and 0.3 kJ/mm. The zigzagged columnar ones were only retained in samples obtained at 0.255 kJ/mm. The fraction of Σ3 boundaries reduced at higher heat input values to 7 and 4%, respectively. The maximum tensile strength was achieved on samples obtained with 0.21 kJ/mm as tested with a tensile axis perpendicular to the deposited wall’s height. More than 100% elongation-to-fracture was achieved when testing the samples obtained at 0.3 kJ/mm (as tested with a tensile axis coinciding with the wall’s height).

## 1. Introduction

Additive manufacturing is a process that allows layer-by-layer assembling of near net-shape articles from even dissimilar materials, which may intermix and even dissolve in each other, thereby creating a transition zone. Transition zones, in fact are always created between the as-deposited material and substrate as well as between successively deposited layers of the same material.

Geometry and surface quality of the articles built using directed energy deposition (DED) methods strongly depend on the deposition process parameters that determine the melted pool temperature, size, and the melted metal viscosity. These parameters, in combination with heat removal conditions, determine the solidification rate, crystallization direction, grain size etc. (i.e., the as-deposited material’s microstructure and mechanical characteristics).

Aluminum bronze is an alloy that possesses both high heat conductivity and fluidity in a melted state, which determine its wide application as a cast alloy [1,2]. Its disadvantages, such as susceptibility to absorption of oxygen and formation of columnar as-cast grains, are factors that limit the use of such an alloy in commercial processes, including additive manufacturing. Oxygen absorption can be effectively eliminated when DED is performed in a vacuum chamber with the use of an electron beam for creating a melted pool and transferring metal from a wire. On the other hand, electron beam deposition in a vacuum is accompanied by an impairing of the heat removal conditions. Since the main heat sinking flow is directed from the melted pool to the substrate and then to the heat-sinking water-cooled table, the cooling rate as well as solidification rate will depend on the distance from the table so that the gradient of structures and phases can be formed. Such an inhomogeneity will enhance the anisotropy of its mechanical characteristics. Therefore, controlling of solidification conditions and heat input is a necessity in growing the near-net-shape and especially thin-walled articles.

Critical analysis of the literature sources devoted to DED allows one to conclude that heat input is a crucial factor that determined the structural evolution and mechanical and functional characteristics of as-deposited metals such as Inconel 625 [3], Al–7Si–0.6Mg [4], Al-Mg alloys [5], Ti6Al4V [6], 2209 duplex stainless steel [7], and aluminum bronze [8,9,10]. In selective laser melting (SLM), the decisive role of the heat input was studied on alloys as follows: the Inconel 718 [11], Ti6Al4V alloy [12], AISI 316 stainless steel and AlSi10Mg alloys [13]. Factoring the importance of heat and mass transfer conditions in EBAM layer deposition, the entire near net shape article formation was reported on the AISI 316 [14,15] and Al-6wt.%Mg alloys [16]. Controlling the EBAM deposition parameters ensured forming thin-walled heat resistant nickel alloy articles with the desired shape [17,18] and microstructure [19], as well as complex-shaped sandwich-panels from Ti6Al4V [20].

Heat input is the most flexible and effective parameter to control heating and melting phenomena in the additive DED process. The higher the heat input, the more intense the heating, the larger the melting pool, and the deeper the remelted layer. On the other hand, too high of a heat input leads to evaporation of low-melting elements such as Mg and Zn, de-alloying, and gas porosity of the as-deposited alloy. Another problem in DED is that large columnar grains are formed in the top part of the articles as compared to those equiaxed ones in the bottom part. Such a grain size distribution can be detrimental from the viewpoint of mechanical characteristics.

In the beginning of deposition, a high cooling rate is achieved when depositing first layers on a cold substrate so that equiaxed grains and even discontinuities, lack of bonding or even hot cracks [21,22] are often formed there. Therefore, increasing the heat input during deposition of the first layers is required to avoid defects. At the same time, reduction of the heat input is required when depositing the top layers due to poor heat removal via reheated underlying layers. Obtaining optimal as-deposited metal structures, and therefore mechanical characteristics, is another problem to be resolved with the DED additive manufacturing. Formation of columnar grains under conditions of either excess heat input or low solidification rate serves to enhance the anisotropy of mechanical characteristics, which is already inherent in them by definition.

Interpass deformation may be one of the most effective methods against the epitaxial growth of large columnar grains [23,24,25,26,27]. However, tuning the heat input may be effective too [28], and would be less tedious and labor consuming. Another remedy may be found in using the heat input that is exponentially changing as a function of the build-up metal height [29].

Varying the heat input as a function of the built-up wall height makes it possible to control cooling and therefore the solidification rate. However, no approach for forming the desired grain structures is guaranteed, especially in materials inclined to form large columnar grains such as nickel superalloys [30], titanium [31], copper [32], bronze [33], nickel titanium [34,35], etc. In connection with this, finding out the limiting maximum and minimum values of the heat input for deposition of each material is an important task. As of now, to the best of our knowledge, we have not found any results published on using heat input as a parameter to control structure and mechanical characteristics in additive manufactured Cu-7.5wt.%Al. The objective of this work was to study the effect of heat input on grain structure and the mechanical characteristics of an EBAM-grown thin aluminum bronze wall.

## 2. Materials and Methods

**Figure 1:**
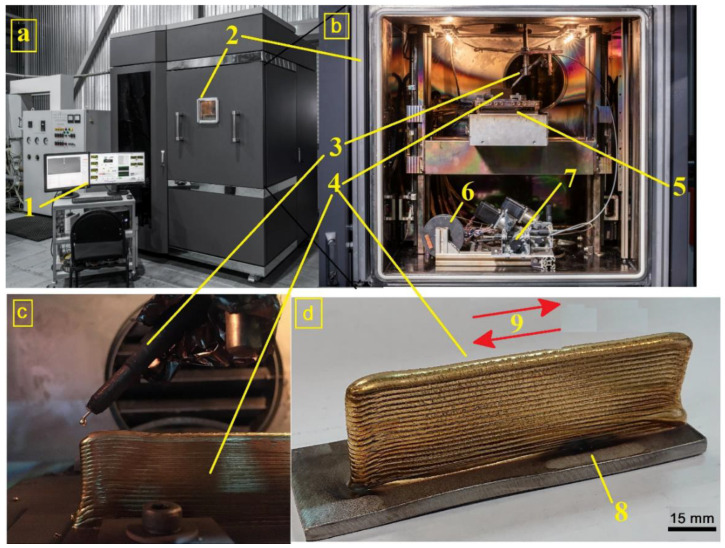
Electron beam wire-feed additive machine (**a**–**c**) and a Cu-7.5wt.%Al wall built (**d**). 1—control panel; 2—vacuum chamber; 3—wire guide; 4—sample printed; 5—water-cooled table; 6—wire reel; 7—wire feeder; 8—stainless steel substrate; 9—layer-by-layer deposition strategy.

An electron beam additive machine was designed and built at the Institute of Strength Physics and Materials Science SB RAS. The deposition was carried out by the melting and transfer of Cu-7.5wt.%Al wire (ESAB) into a melted pool formed by the electron beam on AISI 321 stainless steel substrate in a vacuum chamber with residual pressure 9 × 10^−4^ Pa (Figure 1a–c). A water-cooled table was used to provide effective cooling of the substrate during additive deposition from the wire fed from a reel. The layer-by-layer deposition was performed by moving the table together with the substrate along directions shown by arrows in Figure 1d and then dropping the table before depositing the next layer (Figure 1b).

The heat input was varied during the deposition by varying the electron beam current (Table 1). Several preliminary experiments were carried out to determine the beam current range that allowed building walls suitable for cutting samples for mechanical and microstructural characterization. This range (from 28 to 40 mA) was divided into equal parts with the limiting values used in the next series of experiments (Table 1). When using the beam current values below 28 or above 40 mA, the wall either did not form whatsoever or was overheated and leaked down its sides, respectively.

**Figure 2:**
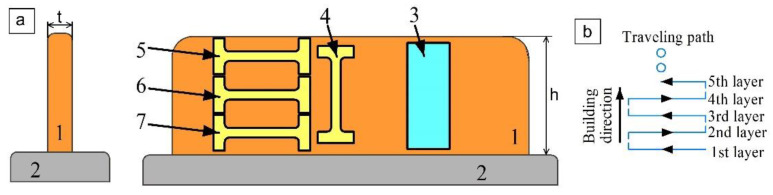
Sample preparation scheme (**a**) and EBAM layer deposition strategy (**b**) 1—Cu-7.5wt.%Al bronze wall; 2—stainless steel substrate; 3—microstructural characterization sample; 4—sample with tensile axis orientation along the wall height; 5–7—samples with tensile axis orientation perpendicular to the wall height (along the layer deposition direction); t—wall thickness; h—wall height.

Samples for characterization of the deposited wall metal for microstructures, phases, and mechanical strength were electrical-discharge (EDM) cut off the wall according to a scheme shown in Figure 2. Plain samples (Figure 2, pos. 3) were intended for microstructural examination after being subjected to corresponding preparation procedures. Samples 5–7 and 4 were cut so that their longitudinal axes were oriented perpendicular and parallel to the tensile machine axis, respectively.

Mechanical characteristics of the samples were determined from tensile tests carried out using a Testsystems 110M-10 (Testsystems, Ivanovo, Russia) tensile machine. The resulting fracture surfaces were examined using a scanning electron microscope (SEM) Microtrac SemTrac mini (Microtrac Inc., Montgomeryville, PA, USA).

Samples intended for microstructural examination were mechanically ground on abrasive 4000-grit paper and then polished using 14,000-grit diamond paste. The polished views were etched in a solution 3% FeCl_3_ + 10% HCL + 100 mL distilled water. The microstructural characterization was performed with the use of an optical microscope Olympus LEXT 4100 (Olympus Corporation, Tokyo, Japan).

An electron backscattering diffraction (EBSD) instrument Nordlys (Oxford Instruments, High Wycombe, UK) mounted in a scanning electron microscope Tescan Mira 3 LMU SEM (TESCAN ORSAY HOLDING, Brno, Czech Republic) was used to study the grain orientation distribution. The EBSD analysis of the obtained data was performed with the use of HKL Channel 5 software (Version 5.12k, Oxford Instruments, High Wycombe, UK). Samples for EBSD were treated using a standard procedure: sandpaper grinding (Mirka), diamond suspension polishing until 0.5 µm (Kemet). Final step was ion polishing at 10 kV for 15 min using SemPrep 2 ion mill (Technoorg Linda, Budapest, Hungary).

## 3. Results

### 3.1. Metallography

**Figure 3:**
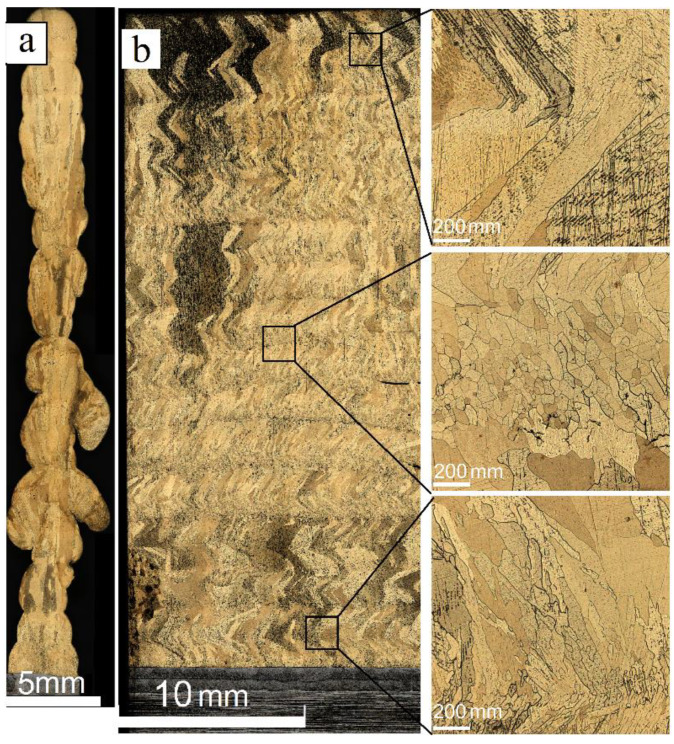
Profile (**a**) and front (**b**) cross section optical views of the Cu-7.5wt.%Al wall obtained at heat input of 0.21 kJ/mm.

The metallographic image obtained from the 35.2 mm of height wall obtained at heat input 0.21 KJ/mm shows the unevenness of the wall thickness, which varied from 1.5 to 4.3 mm along the wall’s height (Figure 3a). Cold laps are observed on the wall sides that could be related to instability of the melt pool and insufficient fusion with the previously deposited layer.

The macrostructure views show at least two different types of grains such as (i) almost equiaxed and (ii) columnar ones with their longest axes oriented at an angle of ~30° with respect to the vertical direction. The columnar grains have a varying thickness so that their thicknesses in the bottom, half-height, and top parts are 100 ± 50 μm, 300 ± 100 μm and 550 ± 125 μm, respectively (Figure 3b).

**Figure 4:**
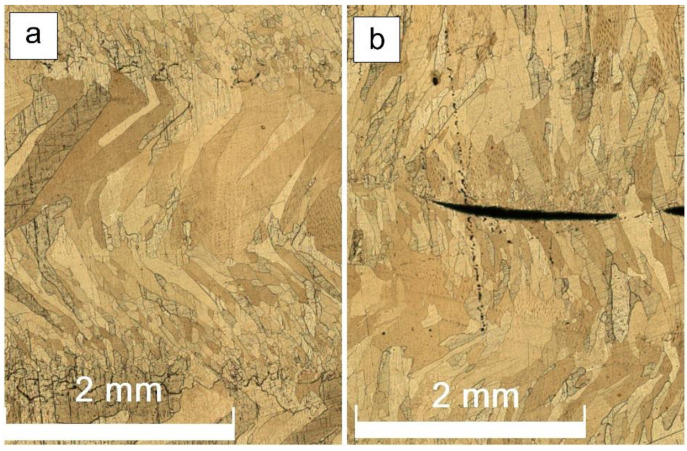
Equiaxed and columnar grain structures (**a**), fusion discontinuities (**b**) in the wall obtained at heat input of 0.21 kJ/mm.

Along with the variable wall’s thickness, there is another problem related to using the low heat input in EBAM from the Cu-7.5wt.%Al wire. The layers composed of small grains were insufficiently remelted when depositing the next layer and, therefore, even some discontinuities were formed (Figure 4b).

**Figure 5:**
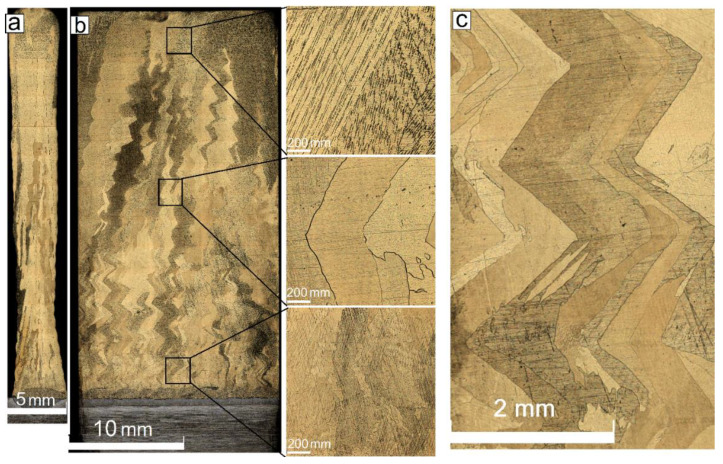
Profile (**a**) and front (**b**,**c**) cross section optical views of the Cu-7.5wt.%Al wall obtained at heat input of 0.255 kJ/mm.

The intermediate heat input level 0.255 kJ/mm allowed obtaining a 33.8 mm height wall characterized by more smooth surfaces without the cold overlaps as well as with its thickness varying from 3 to 4 mm along the height (Figure 5a). Columnar grains still vary with the wall’s height thicknesses and were determined to be 120 ± 50 μm, 400 ± 100 μm, and 700 ± 125 μm in the bottom, half-height, and top parts of the wall, respectively (Figure 5b).

The grains obtained under such an EBAM regime reveal irregular zigzagged grain boundaries formed due to fast solidification and only partial remelting of the underlying grains, which served for epitaxial growth of the new ones oriented with respect to the heat removal and deposition pass direction. Therefore, when reversing the deposition pass direction, the columnar grain orientation is changed too (Figure 5c).

**Figure 6:**
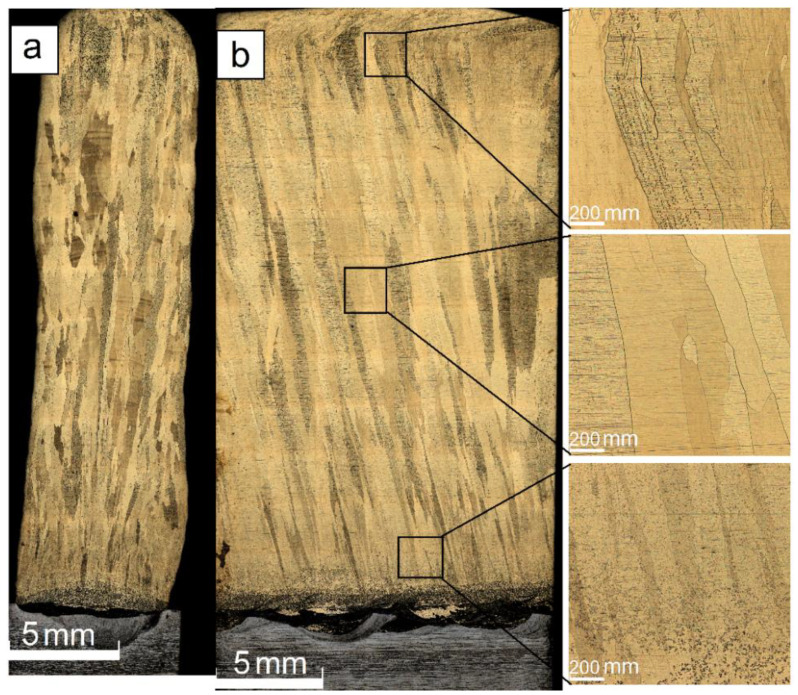
Profile (**a**) and front (**b**) cross section optical views of the Cu-7.5wt.%Al wall obtained at heat input of 0.3 kJ/mm.

EBAM with heat input as high as 0.3 kJ/mm allowed obtaining a 28 mm height wall of thickness varying from 6.9 to 7.3 mm (Figure 6a). Only columnar grains with thicknesses of 150 ± 50 μm, 450 ± 100 μm and 850 ± 125 μm as-measured in the bottom, half-height, and top parts of the wall, have been detected (Figure 6b).

### 3.2. Grain Orientation Maps

**Figure 7:**
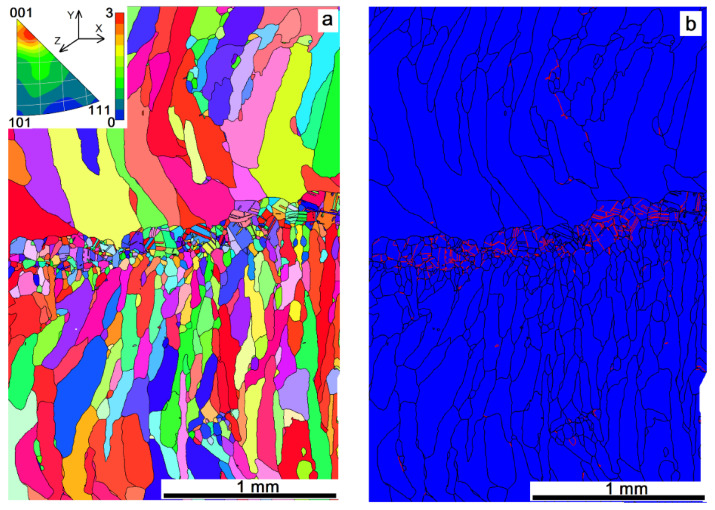
EBSD grain orientation distribution (**a**) and grain boundary types (**b**) in a cross section of the Cu-7.5wt.%Al wall obtained at heat input of 0.21 kJ/mm. Red lines in Figure 7b show the annealing twin boundaries.

Grain orientation maps and boundary misorientation distributions were obtained to evaluate the effect of heat input on the as-deposited microstructures. It was observed that a high number of grains solidified after deposition with heat input 0.21 kJ/mm were oriented with their [001] axes almost along the Z axis (i.e., normal to the sample’s surface (Figure 7a)). Such a preferential orientation distribution corresponds to cubic growth textures commonly formed during solidification from a melted pool formed by a concentrated energy source. However, there are columnar and especially small equiaxed grains with their orientations different from that of the cubic texture. Small equiaxed grains form a layer with high density of annealing twin Σ3 boundaries (Figure 7b).

**Figure 8:**
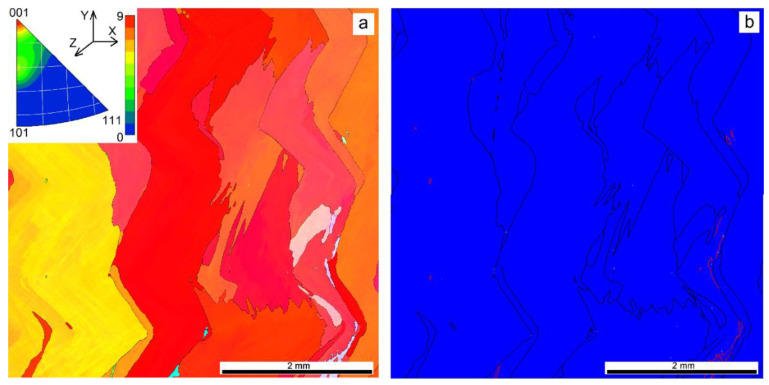
EBSD grain orientation distribution (**a**) and grain boundary types (**b**) in a cross section of the Cu-7.5wt.%Al wall obtained at heat input of 0.255 kJ/mm.

**Figure 9:**
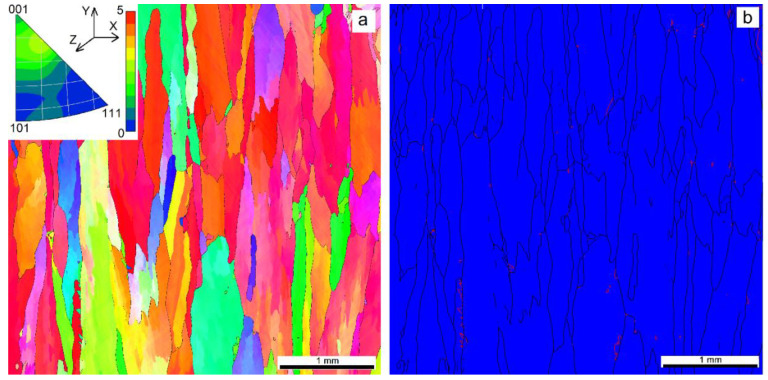
EBSD grain orientation distribution (**a**) and grain boundary types (**b**) in a cross section of the Cu-7.5wt.%Al wall obtained at heat input of 0.3 kJ/mm.

EBAM at heat input 0.255 kJ/mm demonstrated the absence of small grains and more perfect growth texture of the columnar zigzagged ones (Figure 8a). Even higher heat input 0.3 kJ/mm resulted in a reduced number of grains oriented in accordance to the cubic texture. In fact, no preferential orientation was observed in this case, with only small peak corresponding to orientations close to [117]-axis (Figure 9a). A columnar grain microstructure was formed that did not allow finding the fusion boundaries between the successively deposited layers. 

**Figure 10:**
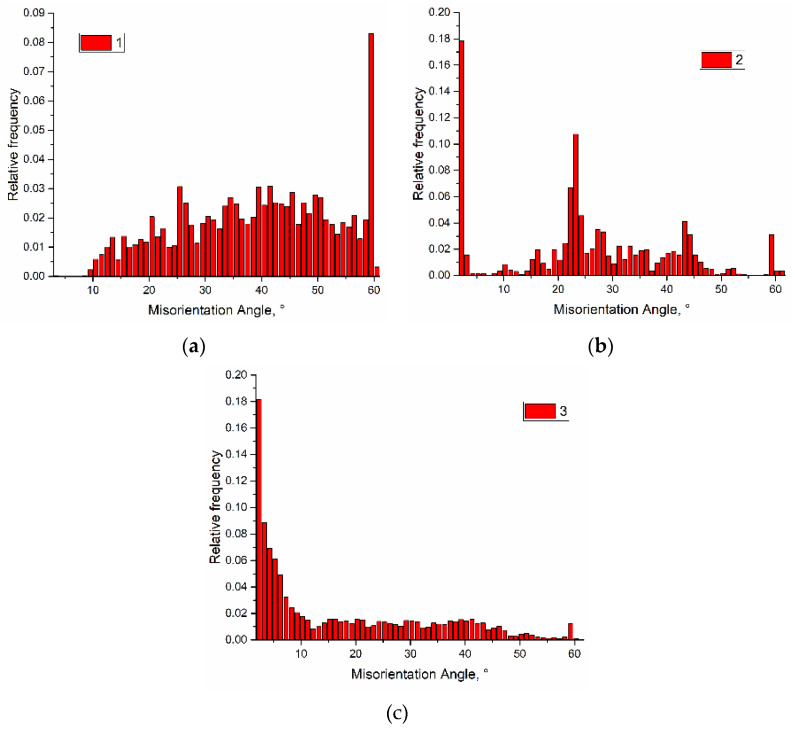
Misorientation angle distribution of grain boundaries for samples printed at 0.21 kJ/mm (**a**), 0.255 kJ/mm (**b**) and 0.3 kJ/mm high (**c**) heat input.

The content of Σ3-type boundaries reduced from 10% to 7% inherent in the sample printed at heat input 0.21 kJ/mm (Figure 10a,b). Further reduction of the annealing twin boundary percentage to 4% was observed (Figure 7b and Figure 10c).

Grain boundary misorientation histograms allow following the grain boundary type evolution as depended on the heat input (Figure 10a–c). With the exception of 10% of Σ3 and lack of the low angle boundaries, almost uniform distribution of the grain boundaries vs. the misorientation angle was observed in a sample obtained with the heat input 0.21 kJ/mm (Figure 10a). The intermediate heat input 0.255 kJ/mm resulted in formation of higher amount of low angle boundaries on the account of high angle ones (Figure 10b). Even more obvious is this redistribution in case of using maximum 0.3 kJ/mm heat input (Figure 10c) when most part of the boundaries were low-angle ones.

### 3.3. Phase Composition

**Figure 11:**
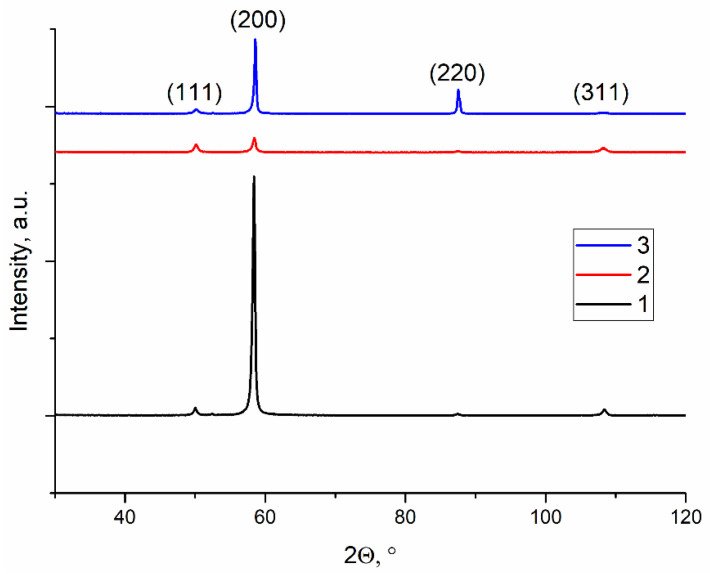
An XRD pattern for the Cu-7.5wt.%Al bronze samples printed at 0.21 kJ/mm (1), 0.255 kJ/mm (2) and 0.3 kJ/mm high (3) heat input.

According to XRD, no phases other than α-Cu solid solution were detected in as-deposited Cu-7.5wt.%Al irrespective of the heat input (Figure 11). Also, the inversion of the (111)/(200) and (220)/(311) peak height ratios shows the presence of the growth texture.

### 3.4. Mechanical Characteristics

**Figure 12:**
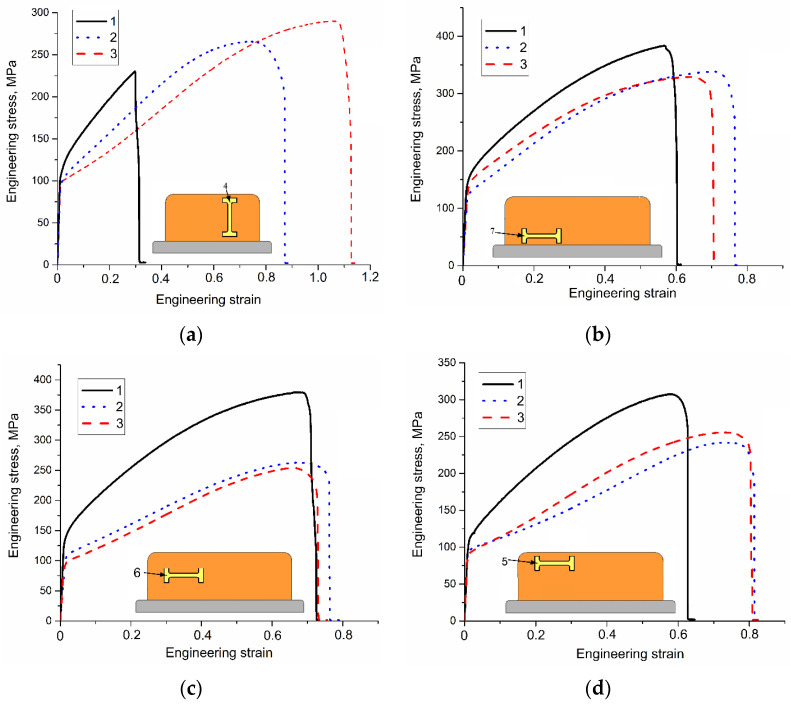
The “stress-strain” curves obtained from tensile tests on Cu-7.5wt.%Al samples 4 (**a**), 7 (**b**), 6 (**c**) and 5 (**d**). EBAM regime: 1—0.21 kJ/mm, 2—0.255 kJ/mm, 3—0.3 kJ/mm.

**Figure 13:**
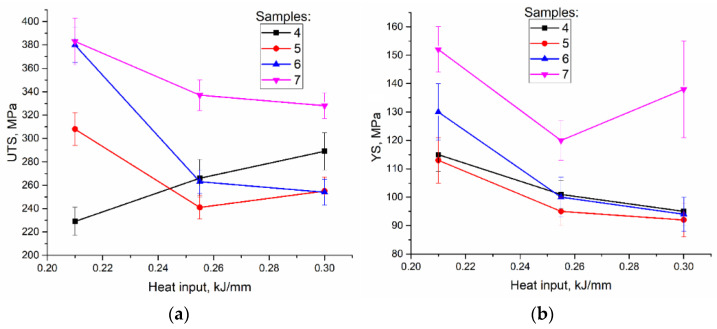
UTS (**a**) and YS (**b**) vs. heat input dependencies.

**Figure 14:**
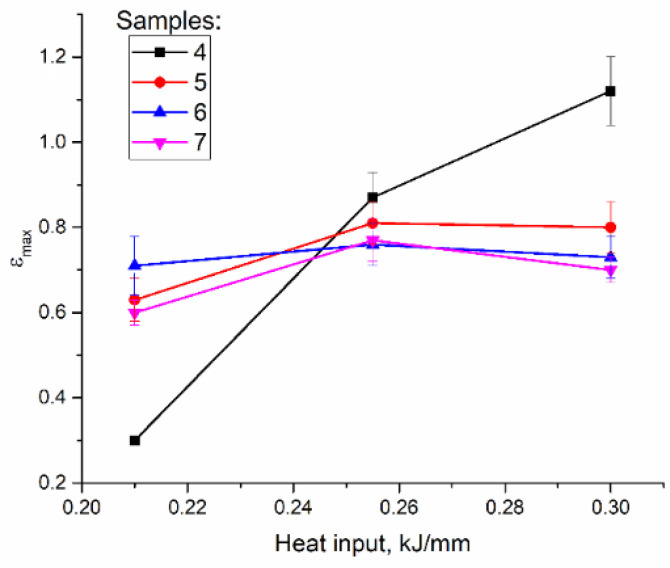
Strain-to-fracture vs. heat input dependencies.

Tensile tests were carried out on samples cut of the EBAM printed walls as shown in Figure 2. Clear differences in tensile behaviors of the samples with tensile axis orientation along the wall height can be observed from Figure 12. The low heat input sample 4 (Figure 12a, curve 1) showed minimal yield stress (YS) and ultimate tensile strength (UTS) values with strain-to-fracture ε_max_ as low as 0.30 ± 0.01 (Figure 13 and Figure 14). Such a quasi-brittle fracture behavior can be explained by the presence of fusion discontinuities, grain size variations (Figure 4b). EBAM at higher heat input levels allowed increasing both UTS and ε_max_, while YS values reduced because of preferential forming the columnar grains whose longest axis were oriented close to the tensile axis. It is known [36] that higher ductility is achieved in tensile tests with that type of columnar grain structures.

Samples 5, 6, and 7 tested with their tensile axes orientation perpendicular to the wall height (Figure 2, pos. 5, 6 and 7) demonstrated different mechanical characteristic dependencies on the wall height and heat input.

First of all, the best UTS (Figure 13a) values were obtained on samples cut off the bottom and half-height parts of the walls built by EBAM at 0.21 kJ/mm (Figure 13a, samples 6, 7). The YS (Figure 13b) values of samples 6 and, especially 5, were notably lower as compared to that of samples 7. At 0.255 kJ/mm and 0.3 kJ/mm the best UTS still belonged to samples 7 despite there was a tendency to reduce the UTS at higher heat input values.

Strain-to-fracture dependencies of samples 5, 6, and 7 on the heat input show a tendency for improving plasticity of all samples at heat input 0.255 kJ/mm as compared to that of at 0.21 kJ/mm and then reducing it at 0.3 kJ/mm (Figure 14). The highest plasticity was achieved at 0.255 kJ/mm on sample 5 (cut off the top part of the wall).

**Figure 15:**
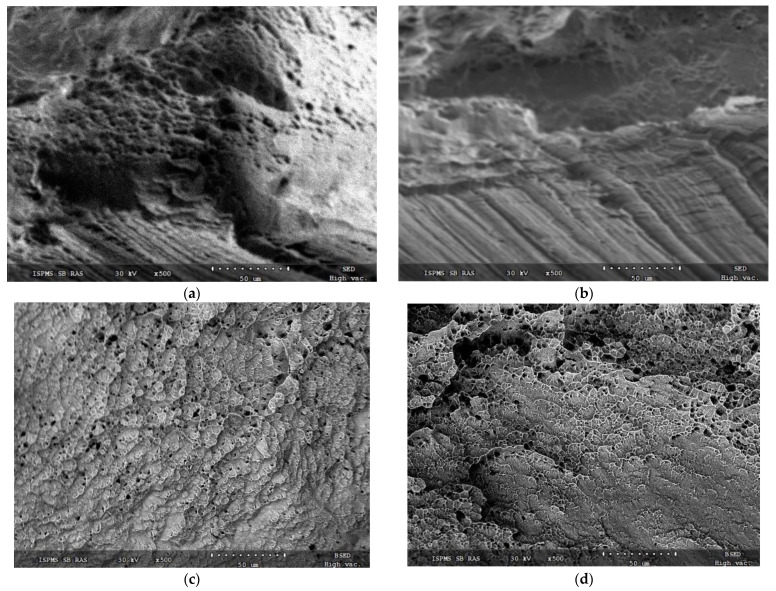
Fracture surface after tension test for samples 4 printed at 0.21 kJ/mm (**a**,**b**), 0.255 kJ/mm (**c**) and 0.3 kJ/mm (**d**) heat input.

Fracture surface of a sample grown at 0.21 kJ/mm can be classified as a surface obtained as a result of mixed viscous/brittle fracture mechanism. Quasi-brittle fracture microgrooves can be observed in Figure 15b along with microcells (Figure 15a), usually inherent with the viscous fracture. Fracture surfaces of samples printed at 0.255 kJ/mm (Figure 15c) and 0.3 kJ/mm (Figure 15d) demonstrate the presence of only viscous fracture microcells and ridges.

**Figure 16:**
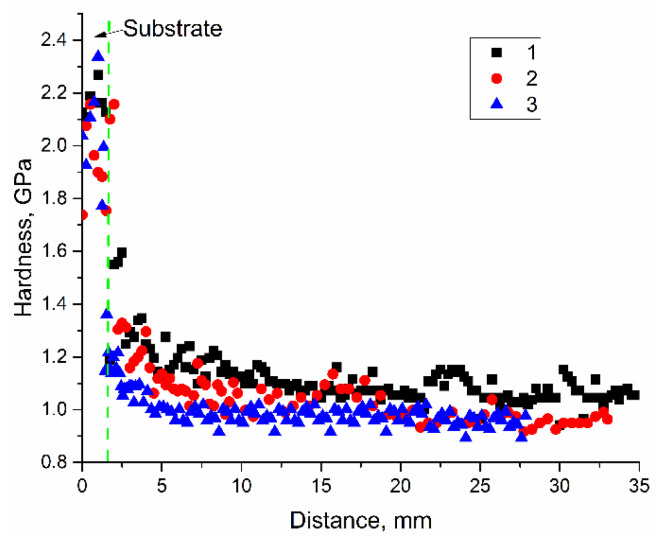
Microhardness profiles as measured along the wall height. EBAM regime: 1, 0.21 kJ/mm; 2, 0.255 kJ/mm; 3, 0.3 kJ/mm.

Microhardness profiles were obtained along the wall height that showed the higher was the heat input, and the lower microhardness was obtained in the Cu-7.5wt.%Al bronze printed (Figure 16) on the stainless-steel substrate. Some transition zone with microhardness higher than that of as-deposited bronze can be observed between the stainless-steel substrate and the wall.

## 4. Discussion

According to the XRD (Figure 11), the heat input values used in this work did not have any effect on the as-deposited metal phase composition. This result seems to be an expected one since the content of aluminum in the alloy is below its solubility limit and corresponds to the α-phase region at the corresponding Al/Cu phase diagram. The most notable changes caused by varying heat input are related to morphology of structures, grain orientation distribution, and grain boundary types. These changes had, however, their effect on mechanical characteristics of the thin wall samples obtained at corresponding heat input levels. Since no dispersion or precipitation hardening was expected in the as-deposited metal, all mechanical characteristics were determined by grain structure and the type of grain boundaries.

Some specific features relating to both geometry and structure of the deposited thin wall samples can be delineated from the above-disclosed results. First of all, the wall’s thickness was increased with the heat input together with corresponding reduction of its height. Higher heat input allowed forming a larger and hotter melted pool, and more metal was transferred from the wire into this pool. As a result, metal solidified in the form of a thicker but lower wall without any cold beads.

**Figure 17:**
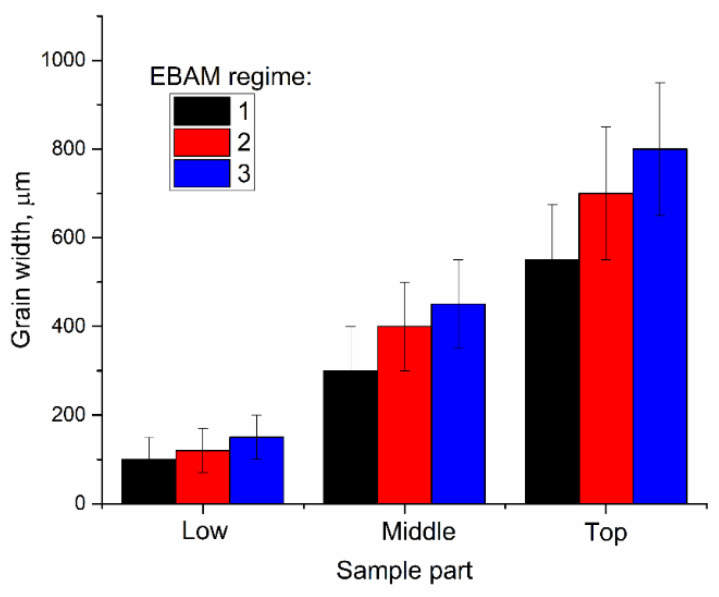
Grain width in different parts of the samples. EBAM regime: (1) 0.21 kJ/mm; (2) 0.255 kJ/mm; (3) 0.3 kJ/mm.

The grain structures also showed the effect of heat input when taking into consideration the grain size and aspect ratio variations along the wall height. It was observed that the grain width as-measured along the *X*-axis (perpendicular to the wall’s height) increased with that wall’s height (*Y*-axis) (Figure 17). Such an observation can be explained by different heat removal conditions existing in the bottom and top parts of the wall during the electron beam wire-feed additive deposition. The bottom part of the wall is close to the water-cooled table and therefore heat removal is more intense there as compared to the top part of the wall, where more heat is accumulated thus contributing to increasing the melted pool size and temperature.

**Figure 18:**
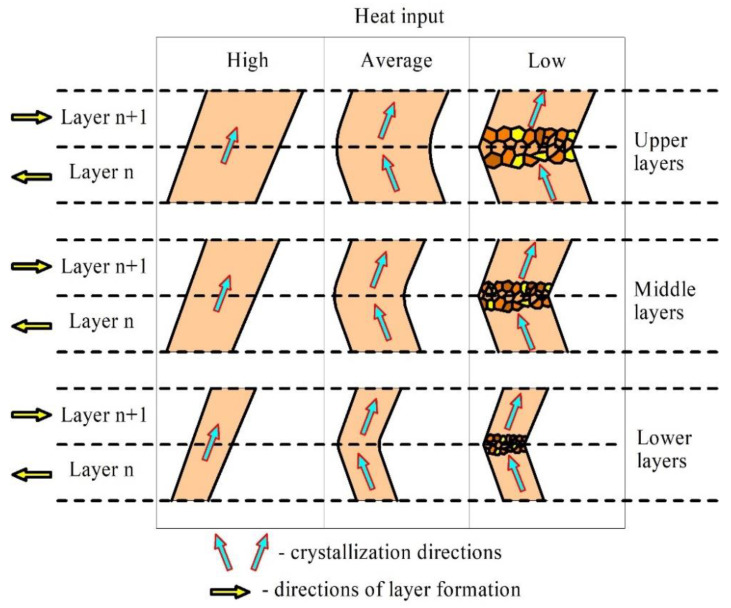
Diagram illustrating the grain growth as depended on the heat input.

Three types of microstructures, which depended on the heat input levels, were observed in the EBAM walls. The first type microstructures are formed at high heat input and described as columnar unidirectional grains grown on the partially melted previously solidified ones according to epitaxial growth mechanism (Figure 18, left part). Columnar high aspect ratio grains slowly grow through the melt so that their orientation coincides with the heat removal irrespective of the deposition pass direction.

**Figure 19:**
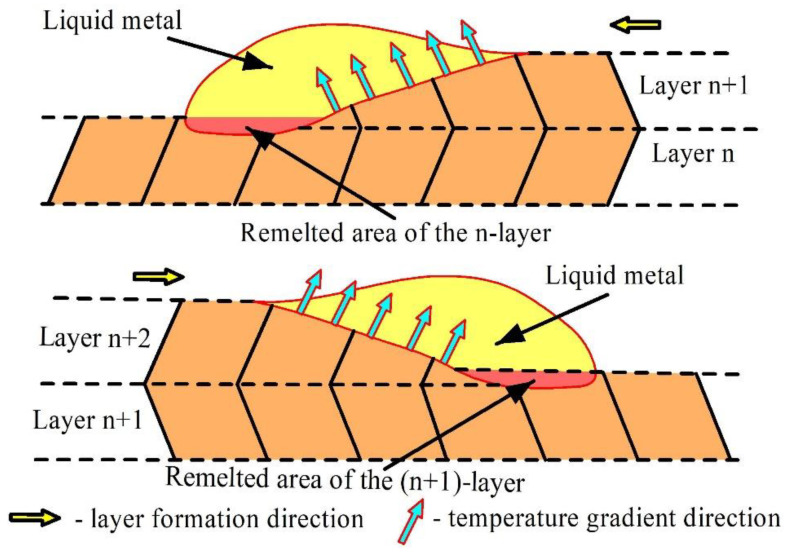
The zigzagged columnar grain structure formation.

The second type of microstructures characterized by zigzagged columnar grains (Figure 18, central part) that were formed at medium heat input conditions when grains grew following the heat removal direction alternating with the deposition pass direction (Figure 19). Such a zigzagged grain growth mechanism was reported elsewhere [37].

Along with columnar grains, the third type microstructures contained layers of small almost equiaxed grains with high-angle and annealing twin boundaries that were formed due to low heat input and fast solidification of the first metal portions transferred from the wire metal onto a thin partially melted and poorly heated layer of previously deposited grains.

Further portions of metal transferred more heat to this poorly preheated melted pool and thus increase its temperature, reduce the solidification rate and thus allow for the growing of columnar grains during the deposition pass (Figure 18, right part).

At 0.21 kJ/mm solidification rate was high enough to cause formation of smaller equiaxed grains in the fusion zone between successively deposited layers. These equiaxed grain layers contain both discontinuities and high amount of high-angle, as well as annealing twin boundaries that act as effective dislocation barriers and thus lead to fast strain localization in such a region with ensuing quasi-brittle fracture.

Increasing the heat input resulted in reducing the number of twin boundaries first to 7% at 0.225 kJ/mm and then to 4% at 0.3 kJ/mm. It is suggested that such a result is determined by solidification rate which is reduced at higher heat input when large columnar grain slowly grow from the partially melted metal of the previously deposited layer. Structural changes that occur at increased heat input resulted in decreasing both strength characteristics with simultaneous improving the plasticity. It can be observed from Figure 16 that the grain width is increased with the heat input thus providing less dislocation barriers. Also, there is a tendency that more low-angle grain boundaries are formed with increasing the heat input (i.e., less effective dislocation barriers and longer dislocation mean free path provide higher plasticity).

The lowest strength was achieved on samples 4 obtained at 0.21 kJ/mm and oriented with their tensile axis along the wall’s height. Such a result is provided by poor welding between successively deposited layers and the presence of discontinuities.

The zigzagged (0.225 kJ/mm) and columnar (0.3 kJ/mm) grains provided some strength improvement due to more perfect fusion boundaries. This, however, did not serve to improve the plasticity of samples 4 where tensile axis was parallel with the wall’s height.

Strength characteristics of samples oriented with their tensile axis along direction to the thin wall’s height depended on this height so that the maximum strength was observed on samples 5 and 6 from the bottom and half-height parts, respectively, of the wall deposited at 0.21 kJ/mm.

Mean microhardness (Figure 16) is reduced as the heat input increased for the same reasons as outlined above (i.e., due to formation of larger grains and fewer high-angle boundaries).

## 5. Conclusions

In this study, the effect of heat input on geometry, grain structure, and mechanical characteristics of electron beam wire-feed additive manufactured Cu-7.5wt.%Al wall has been investigated. The interplay of two factors, namely (i) heat input and (ii) heat removal, was responsible for structure formation during the deposition.

Low heat input resulted in a relatively “cold” deposition and formations of zigzagged columnar grains with small equiaxed ones in the interlayer fusion zones due to local fast cooling of less heated metal and fast solidification. Some samples contained discontinuities in this zone. The maximum 10% fraction of annealing twin boundaries was detected in this case. All of this provided low strength and ductility characteristics of the as-deposited samples 4 oriented along the wall’s height. Strain-to-fracture dependencies on the heat input show that plasticity of these samples grows with the heat input from 0.3 at 0.21 kJ/mm to 0.82 (0.225 kJ/mm) and 1.12 (0.3 kJ/mm). Simultaneously, the ultimate tensile strength is absolute minimum at 0.21 kJ/mm and then is increased by 26 and 29% at 0.225 and 0.5 kJ/mm, respectively.

Increasing the heat input from 0.21 kJ/mm to 0.3 kJ/mm leads to formation of columnar grains with less fraction of high-angle boundaries, including the Σ3 ones whose fraction reduced from 10% to 7% and 4%, respectively. Another finding is that the grain width in bottom, half-height, and top parts of the wall increased by 20–50%, 33–50%, and 27–45%, respectively.

Mechanical tensile strength characteristics of samples with tensile axis perpendicular to the wall’s height showed some reduction of as the heat input increased from 0.21 to 0.3 kJ/mm such that ultimate tensile samples cut of the bottom, half-height, and top part of the wall reduced by 21%, 28%, and 18%, respectively.

## Figures and Tables

**Table 1:** EBAM process parameters.

Regime	Beam Current, mA	Layer Deposition Rate, mm/min	Accelerating Voltage, kV	Heat Input, kJ/mm
1	28	240	30	0.21
2	34	240	30	0.255
3	40	240	30	0.3

## Data Availability

Data will be made available based on request to the authors.
